# The Clinical Factors Related to the Efficacy of Ozoralizumab in Patients with Rheumatoid Arthritis

**DOI:** 10.3390/jcm15218603

**Published:** 2026-11-04

**Authors:** Katsuaki Kanbe, Ayako Kubota

**Affiliations:** 1 Nippori Orthopaedics and Rheumatic Clinic, Tokyo 116-0014, Japan; 2 Toho University Omori Medical Center, Tokyo 143-8541, Japan

**Keywords:** ozoralizumab, rheumatoid arthritis, clinical factors, CCP, disease duration

## Abstract

**Background/Objectives:** Ozoralizumab, a tumor necrosis factor (TNF) blocker, was launched in Japan in 2022. However, there is no evidence to determine whether this biological agent is the best choice for patients with rheumatoid arthritis (RA). To investigate the clinical factors related to the efficacy of ozoralizumab, we analyzed 100 patients treated with ozoralizumab in real-world practice. **Methods:** 100 patients with RA treated with ozoralizumab (female, *n* = 87; male, *n* = 13), with a mean age of 61.8 years, and a mean disease duration of 10.7 years. Methotrexate (MTX) and prednisolone (PSL) were used by 49% and 10% of patients, respectively. The cohort included biologic-naïve patients (*n* = 39) and patients who switched from another biologic agent (*n* = 61). Rheumatoid factor (RF) and anti-cyclic citrullinated peptide (anti-CCP) positivity rates were 91% and 74%, respectively. We analyzed clinical factors associated with Disease Activity Score 28 C-reactive protein (DAS28-CRP) at 24 weeks and assessed remission, treatment continuation, and adverse-event rates. **Results:** DAS28-CRP decreased from 4.38 at baseline to 2.59 at 24 weeks. The remission rate was 59% at 24 weeks. DAS28-CRP at 24 weeks correlated with the anti-CCP titer in the MTX-positive group. Disease duration was correlated with DAS28-CRP at 24 weeks in the MTX-negative and biologic-naïve groups. Remission at 24 weeks was correlated with disease duration. Adverse events occurred in 9% of patients, and all cases improved. **Conclusions:** The efficacy and safety of ozoralizumab were confirmed in 100 patients with RA. Anti-CCP and disease duration are clinical factors that may predict the efficacy of ozoralizumab in RA. Therefore, a high anti-CCP titer or early RA may indicate the efficacy of ozoralizumab in real-world RA practice.

## 1. Introduction

Rheumatoid arthritis (RA) treatment has significantly developed over the past 20 years. Methotrexate (MTX) is recommended as first-line anchor therapy following diagnosis in both the Japan College of Rheumatology (JCR) clinical practice guidelines [1] and the European League Against Rheumatism (EULAR) recommendations [2]. In phase II, a biologic agent or Janus kinase (JAK) inhibitor is considered when MTX treatment is insufficient in patients with RA [1]. However, in clinical practice, it is difficult for physicians to select the most effective drug for each patient.

Anti-tumor necrosis factor (TNF)-α therapies have shown high therapeutic efficacy in RA, and five biologics are currently available: infliximab, etanercept, adalimumab, golimumab, certolizumab [3], as well as three biosimilar drugs for TNF blockade. Ozoralizumab (OZR) is a recent anti-TNF biologic agent that was approved in Japan in September 2022. The next-generation anti-TNF-α single-domain antibody ozoralizumab is a recombinant humanized trivalent NANOBODY^®^ compound consisting of two anti-TNF-α NANOBODY^®^ (variable region domain of heavy-chain antibodies) molecules and one anti-human serum albumin (HSA) NANOBODY^®^ molecule [4]. It has a molecular weight of 38 kDa, which is approximately one-quarter that of representative existing anti-TNF-α IgG antibodies. The HSA-binding domain contributes to the long serum half-life of ozoralizumab [5]. Its efficacy has been demonstrated in domestic clinical trials, including the OHZORA and NATSUZORA studies [6,7]. However, the clinical factors related to the efficacy of ozoralizumab, especially in real-world practice, including switching cases from other biologics and monotherapy, are unknown. We investigated factors predictive of efficacy in 100 patients treated with ozoralizumab for RA.

## 2. Materials and Methods

We analyzed 100 patients with RA (87 women and 13 men) treated by ozoralizumab (Taisho Pharmaceutical Co., Ltd., Tokyo, Japan) with a mean age of 61.8 years (range, 24–87 years) and a mean disease duration of 10.7 years (range, 0.3–50 years). This work was the retrospective observational study, and the follow-up period was 24 months. MTX and prednisolone (PSL) were used by 49% and 10% of patients, respectively. Thirty-nine patients were biologic-naïve, and 61 were switch cases. Rheumatoid factor (RF) and anti-cyclic citrullinated peptide (anti-CCP) antibody positivity rates were 91% and 74%, respectively. Clinical factors associated with Disease Activity Score 28 C-reactive protein (DAS28-CRP) at 24 weeks, as well as remission, continuation, and adverse-event rates, were statistically analyzed [8].

All patients were diagnosed with RA according to the following criteria set by the American College of Rheumatology (ACR)/European League Against Rheumatism (EULAR) 2010 RA classification criteria [9]. Inclusion criteria was active disease for ≥3 months despite previous use of disease-modifying antirheumatic drugs (DMARDs) in biologic-naïve patients (*n* = 39) or with biologic agents in switch patients (*n* = 61). Patients who switched from other biologic treatments had previously received golimumab (*n* = 31), abatacept (*n* = 12), sarilumab (*n* = 9), etanercept (*n* = 4), certolizumab pegol (*n* = 2), infliximab (*n* = 1), tocilizumab (*n* = 1), or adalimumab (*n* = 1). All patients provided their written informed consent to participate in the study, which was approved by the ethics committee of our hospital (No. 50001). This study was conducted in accordance with the principles of the Declaration of Helsinki. Patients were screened for latent and active tuberculosis. Patients infected with hepatitis B or hepatitis C virus were excluded. The baseline DAS28-CRP level was 4.38 ± 0.65 (Table 1). The mean anti-CCP titer was 140.2 ± 242.1 (positive rate 74%). The mean RF was 136.8 ± 226.1 (positive rate, 91%). Patients (*n* = 100) received ozoralizumab 30 mg monthly. The mean doses of MTX and prednisolone (PSL) at baseline were 6.4 ± 2.3 mg/week (*n* = 49) and 3.75 ± 1.34 mg/day (*n* = 10), respectively. The mean doses of MTX and prednisolone (PSL) at baseline were 3.2 ± 3.6 mg/week (0–12 mg/weeks, *n* = 100) and 0.38 ± 1.2 mg/day (0–5 mg/day, *n* = 100), respectively. The doses of MTX and PSL were changed by the efficacy or side effects during treatment. According to the criteria established by Steinbrocker et al. [10], patients were classified as stage I (*n* = 16), stage II (*n* = 43), stage III (*n* = 16), stage IV (*n* = 25), and class 1 (*n* = 12), class 2 (*n* = 69), or class 3 (*n* = 19). Clinical efficacy was evaluated by comparing DAS28-CRP at 24 weeks with the baseline value. Correlations of DAS28-CRP at 24 weeks with anti-CCP, RF, and disease duration were analyzed. Clinical remission was defined by using DAS28-CRP < 2.6 at 24 weeks. These correlations were also analyzed in the MTX-positive and MTX-negative groups and the biologic-naïve and switch groups. A logistic regression analysis was performed using baseline clinical factors such as age, disease duration, RF, anti-CCP, MTX, and PSL. The continuation rate was estimated using the Kaplan–Meier method for the overall cohort and for groups stratified by MTX use and age 65 years.

### Statistical Analysis

The Wilcoxon signed-rank test was used to compare nonparametric continuous variables, including DAS28-CRP and serum RF levels, between baseline and 24 weeks. A logistic regression analysis and Spearman correlation analyses were performed to assess factors associated with DAS28-CRP at 24 weeks using Stat-Flex (ver. 7.0). Statistical significance was set at *p* < 0.05. The log-rank test was used to compare continuation rates between the MTX-positive and MTX-negative groups and between the over- and under-65-year-olds.

## 3. Results

**Figure 1:**
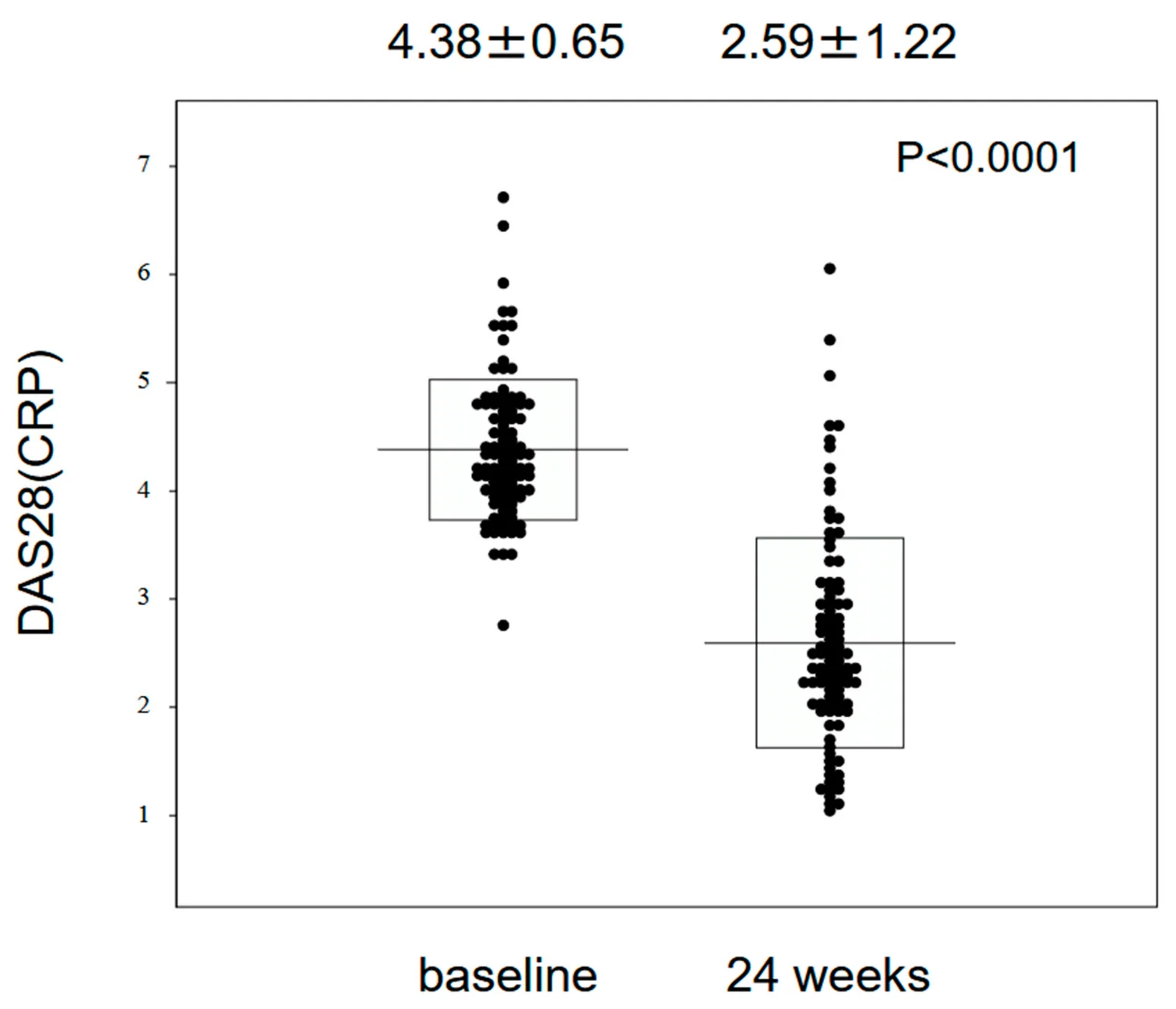
Comparison of DAS28-CRP between baseline and 24 weeks after ozoralizumab treatment. DAS28-CRP, Disease Activity Score in 28 joints using C-reactive protein.

**Figure 2:**
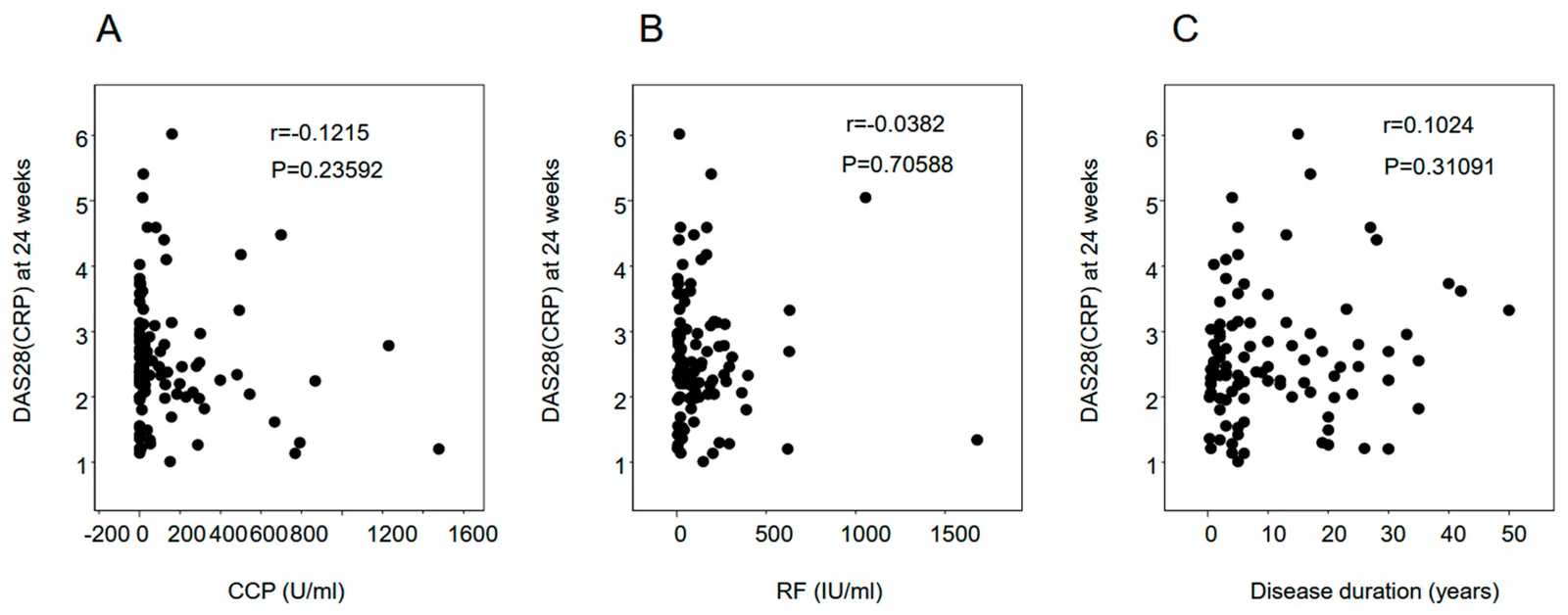
Correlations of DAS28-CRP at 24 weeks with anti-CCP (**A**), RF (**B**) and disease duration (**C**) in the total cohort (*n* = 100). Anti-CCP, cyclic citrullinated peptide; DAS28-CRP, Disease Activity Score in 28 joints using C-reactive protein; RF, rheumatoid factor.

DAS28-CRP decreased significantly from 4.38 ± 0.65 at baseline to 2.59 ± 1.22 at 24 weeks (*p* < 0.0001; Figure 1). The DAS28-CRP remission rate at 24 weeks was 59% (*n* = 91). Nine patients were discontinued at 24 weeks, including insufficient effect (*n* = 5) and side effects, including fatigue (*n* = 1) and skin rash (*n* = 3). The remission rate was calculated as 59 out of 100 baseline patients at 24 weeks. RF levels decreased significantly from 136.8 ± 226.1 at baseline to 106.1 ± 142.3 at 24 weeks (*p* = 0.03341). In the overall cohort, DAS28-CRP at 24 weeks was not significantly correlated with anti-CCP titer (*p* = 0.23592, r = −0.1215), RF level (*p* = 0.70588, r = −0.0382), or disease duration (*p* = 0.31091, r = 0.1024; Figure 2).

Regarding baseline DAS28(CRP), in the MTX-positive group, the correlation and *p*-value between DAS28(CRP) at baseline and CCP was r = −0.0322, *p* = 0.82792; RF was r = 0.0574, *p* = 0.69513; and D.D. was r = −0.39, *p* = 0.00561. In the MTX-negative group, the correlation and *p*-value between DAS28(CRP) at baseline and CCP was r = 0.1559, *p* = 0.28481; RF was r = −0.1121, *p* = 0.43361; and D.D. was r =−0.0231, *p* = 0.87238. In the naïve group, the correlation and *p*-value between DAS28(CRP) at baseline and CCP was r = 0.0913, *p* = 0.58041; RF was r = −0.0707, *p* = 0.66873; and D.D. was r = 0.0639, *p* = 0.69908. In the switch group, the correlation and *p*-value between DAS28(CRP) at baseline and CCP was r = 0.0882, *p* = 0.51038; RF was r = −0.006, *p* = 0.96329; and D.D. was r = −0.1459, *p* = 0.26185. Therefore, there is no significant correlation in baseline DAS28(CRP) except for a significant correlation between DAS28(CRP) at baseline and disease duration in MTX-positive groups.

**Figure 3:**
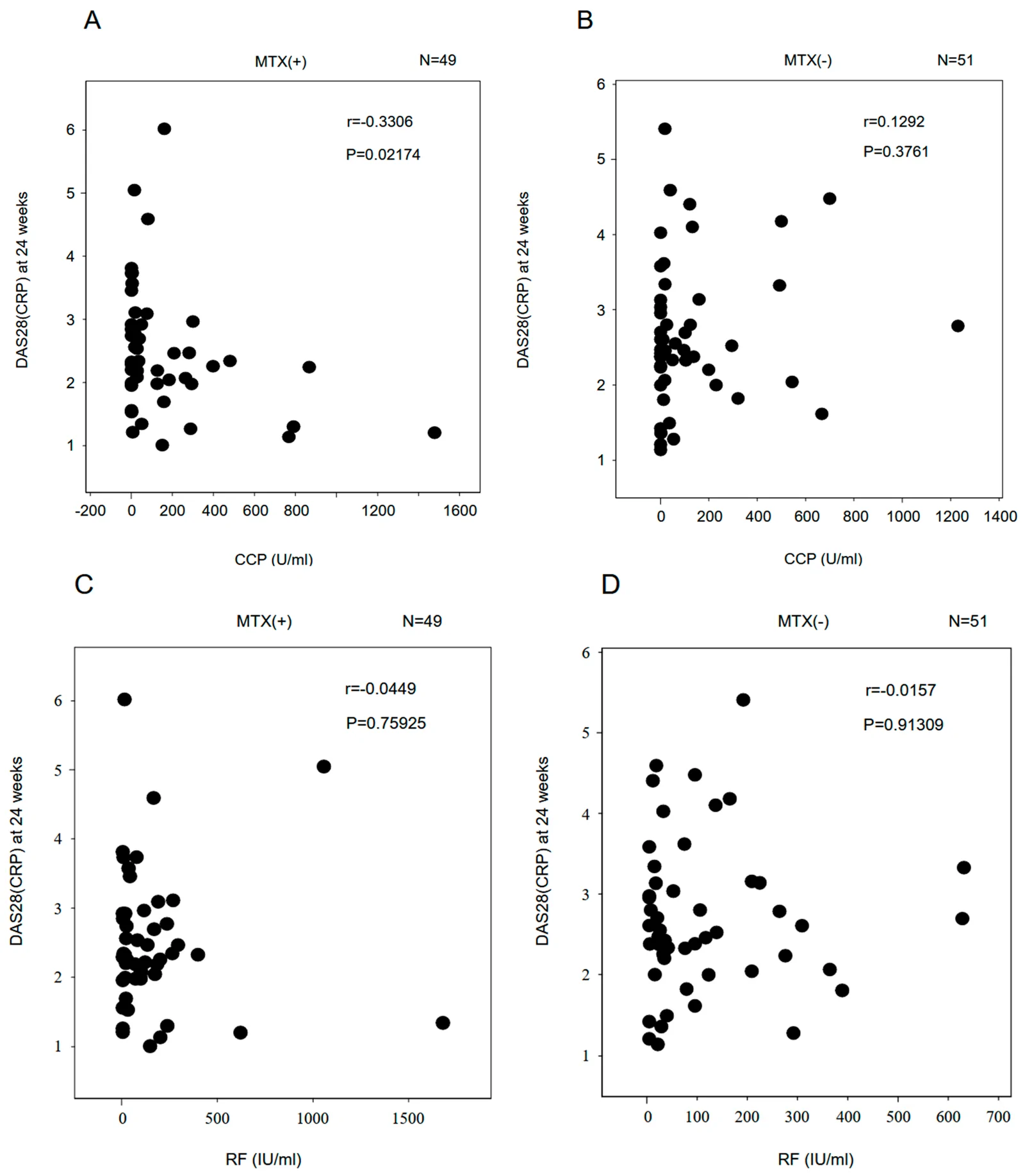

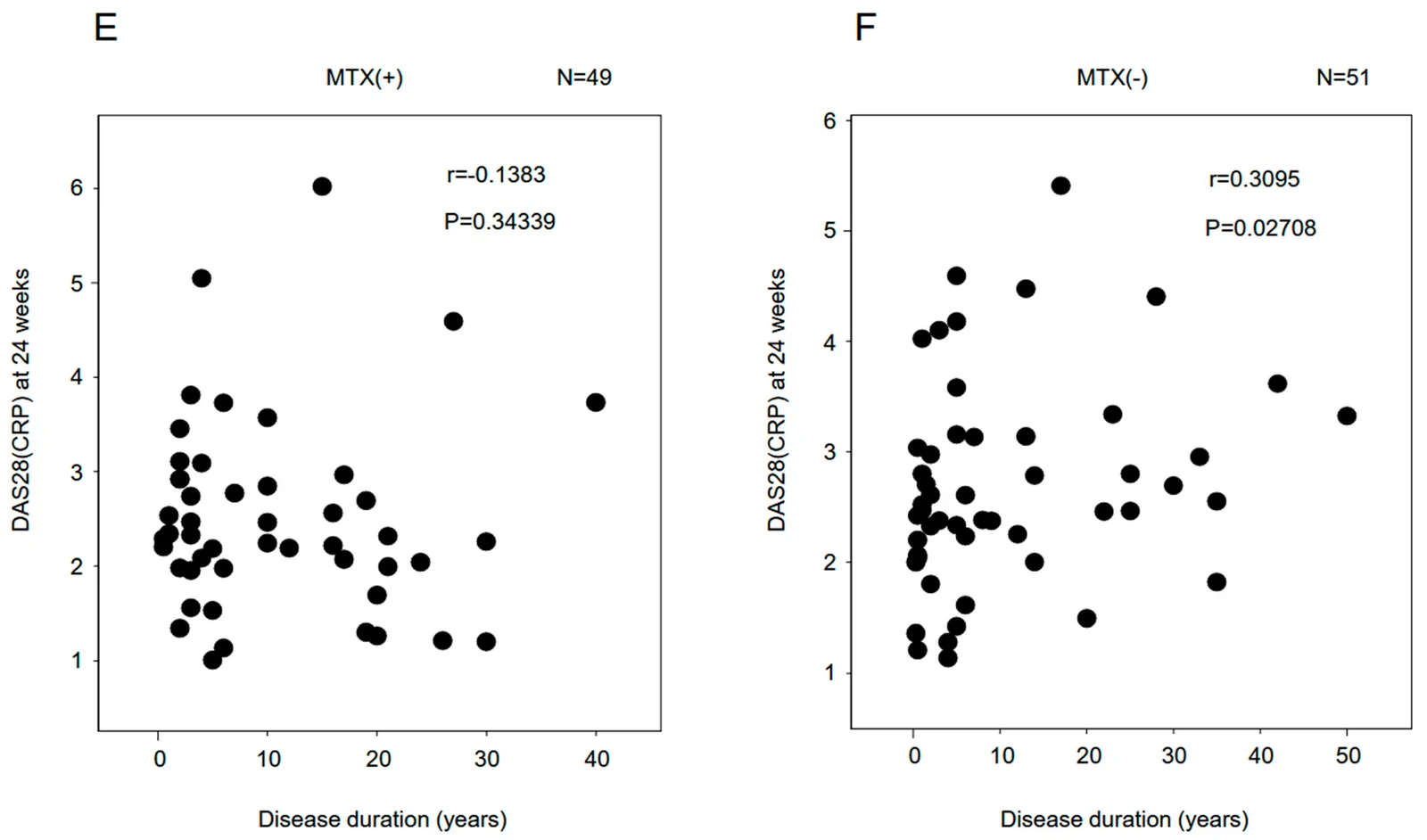
Correlation of DAS28-CRP at 24 weeks with anti-CCP in MTX-positive (**A**) and MTX-negative (**B**) groups. Correlation of DAS28-CRP at 24 weeks with RF in the MTX-positive (**C**) and MTX-negative (**D**) groups. Correlation of DAS28-CRP at 24 weeks with disease duration in the MTX-positive (**E**) and MTX-negative (**F**) groups. Anti-CCP, anti-cyclic citrullinated peptide; DAS28-CRP, Disease Activity Score in 28 joints using C-reactive protein; MTX, methotrexate; RF, rheumatoid factor.

In the MTX-positive group, DAS28-CRP at 24 weeks was significantly correlated with anti-CCP titer (*p* = 0.02174, r = −0.3306). No significant correlation was observed in the MTX-negative group (*p* = 0.3761, r = 0.1292; Figure 3A,B). DAS28-CRP at 24 weeks was not significantly correlated with RF level in either the MTX-positive group (*p* = 0.75925, r =−0.0449) or the MTX-negative group (*p* = 0.91309, r = −0.0157; Figure 3C,D). DAS28-CRP at 24 weeks was significantly correlated with disease duration in the MTX-negative group (*p* = 0.02708, r =0.3095), but not in the MTX-positive group (*p* = 0.34339, r = −0.1383; Figure 3E,F).

**Figure 4:**
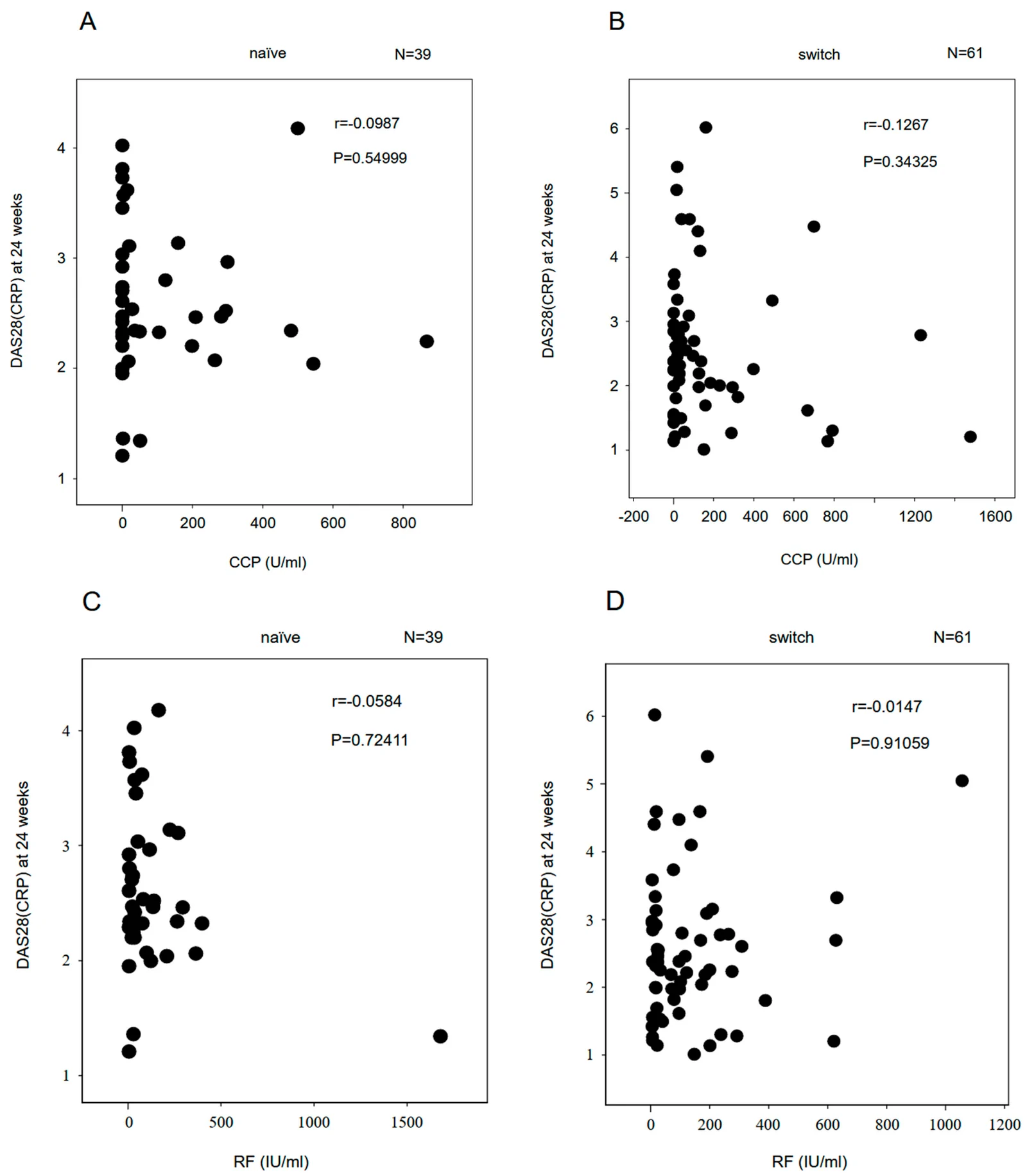

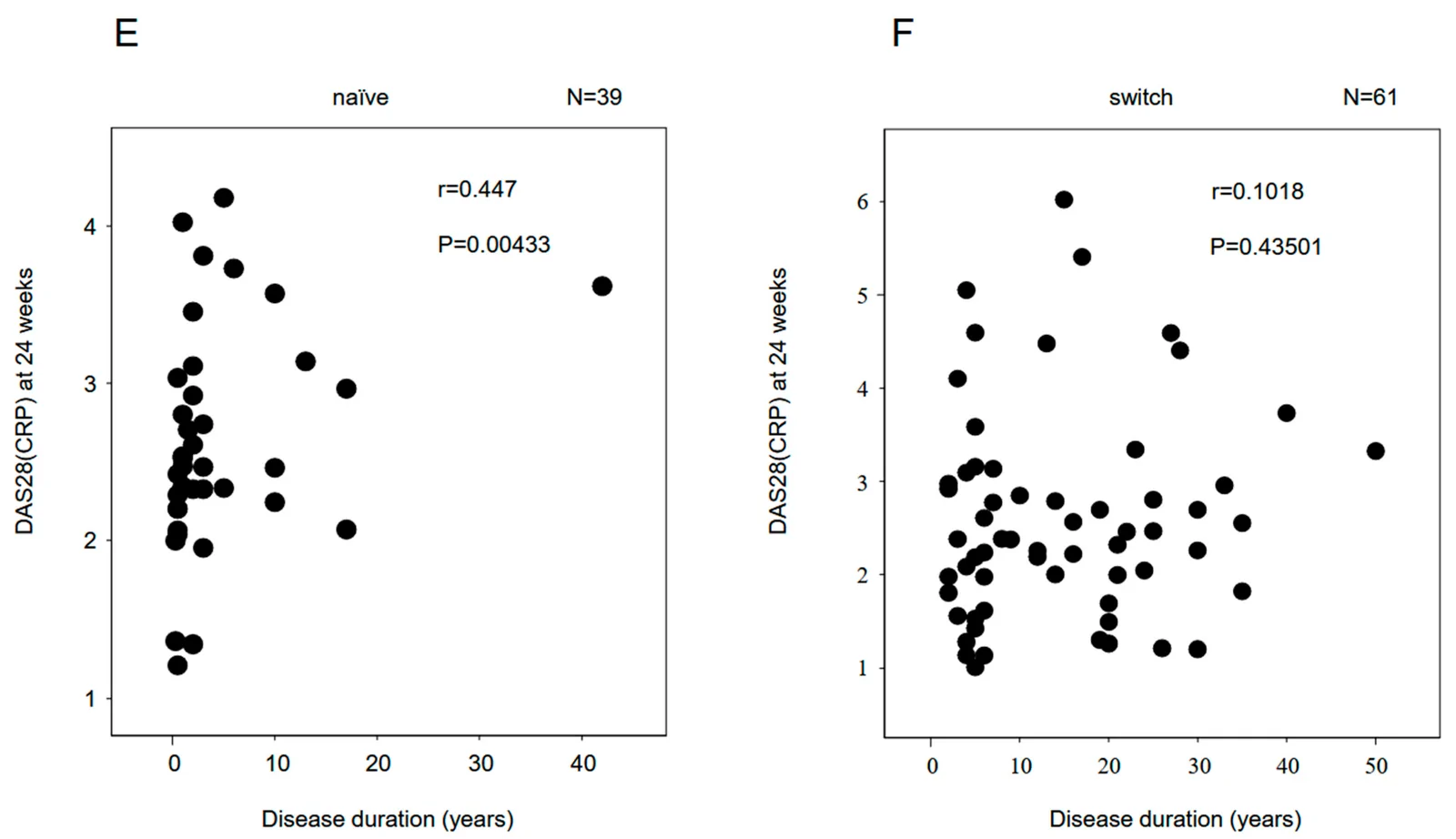
Correlation of DAS28-CRP at 24 weeks with anti-CCP in the biologic-naïve (**A**) and switch (**B**) groups. Correlation of DAS28-CRP at 24 weeks with RF in the biologic-naïve (**C**) and switch (**D**) groups. Correlation of DAS28-CRP at 24 weeks with disease duration in the biologic-naïve (**E**) and switch (**F**) groups. Anti-CCP, anti-cyclic citrullinated peptide; DAS28-CRP, Disease Activity Score in 28 joints using C-reactive protein; RF, rheumatoid factor.

DAS28-CRP at 24 weeks was not significantly correlated with anti-CCP titer in either the biologic-naïve group (*p* = 0.54999, r = −0.0987) or the switch group (*p* = 0.34325, r = −0.1267; Figure 4A,B). No significant correlation with RF level was observed in either the biologic-naïve group (*p* = 0.72411, r = −0.0584) or the switch group (*p* = 0.91059, r = −0.0147; Figure 4C,D). DAS28-CRP at 24 weeks was significantly correlated with disease duration in the biologic-naïve group (*p* = 0.00433, r =0.447), but not in the switch group (*p* = 0.43501, r = 0.1018; Figure 4E,F).

**Figure 5:**
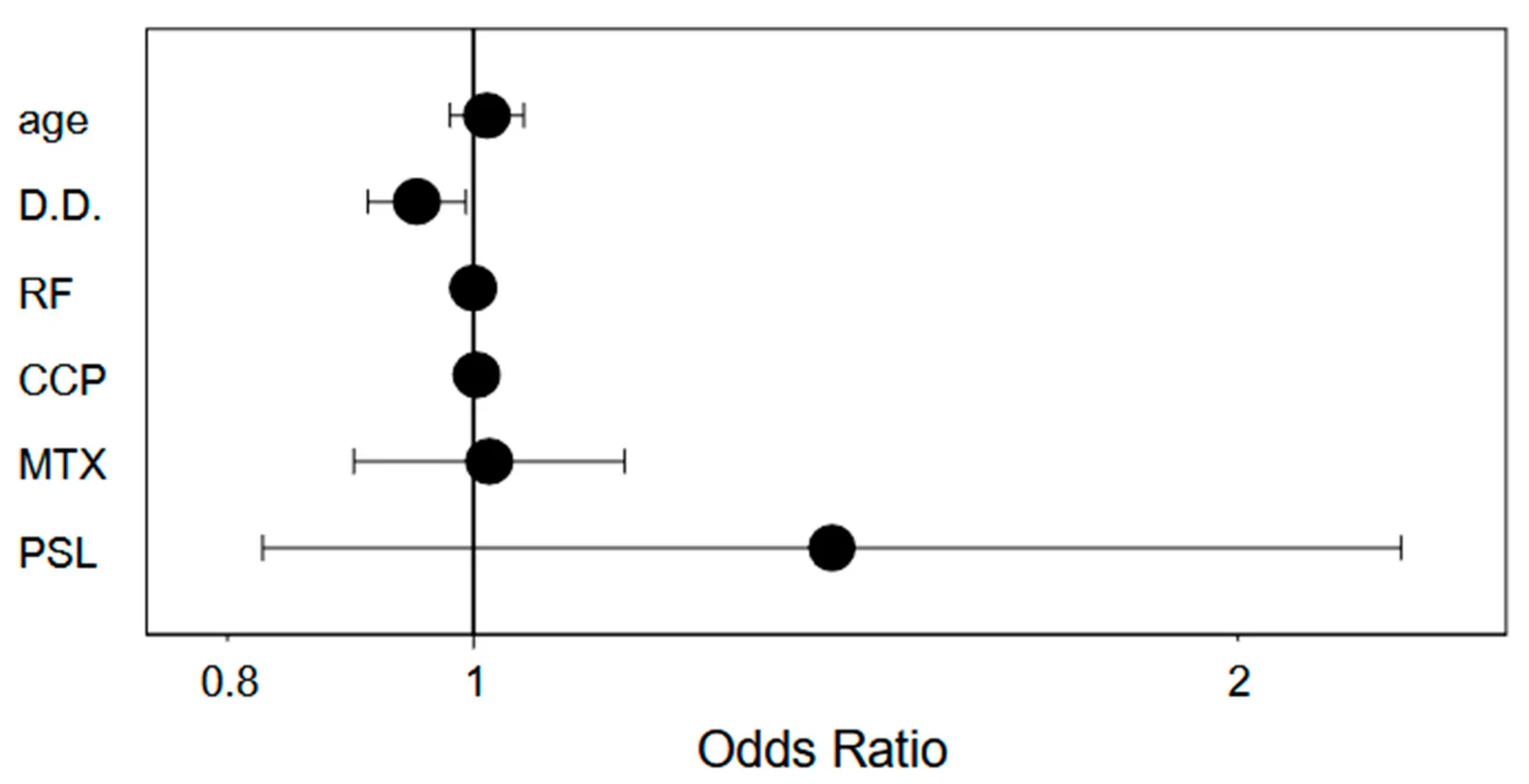
A logistic regression analysis of the associations of remission with age, disease duration, RF, anti-CCP titer, MTX, and PSL. Anti-CCP, anti-cyclic citrullinated peptide; MTX, methotrexate; PSL, prednisolone; RF, rheumatoid factor; ROC, receiver operating characteristic.

**Figure 6:**
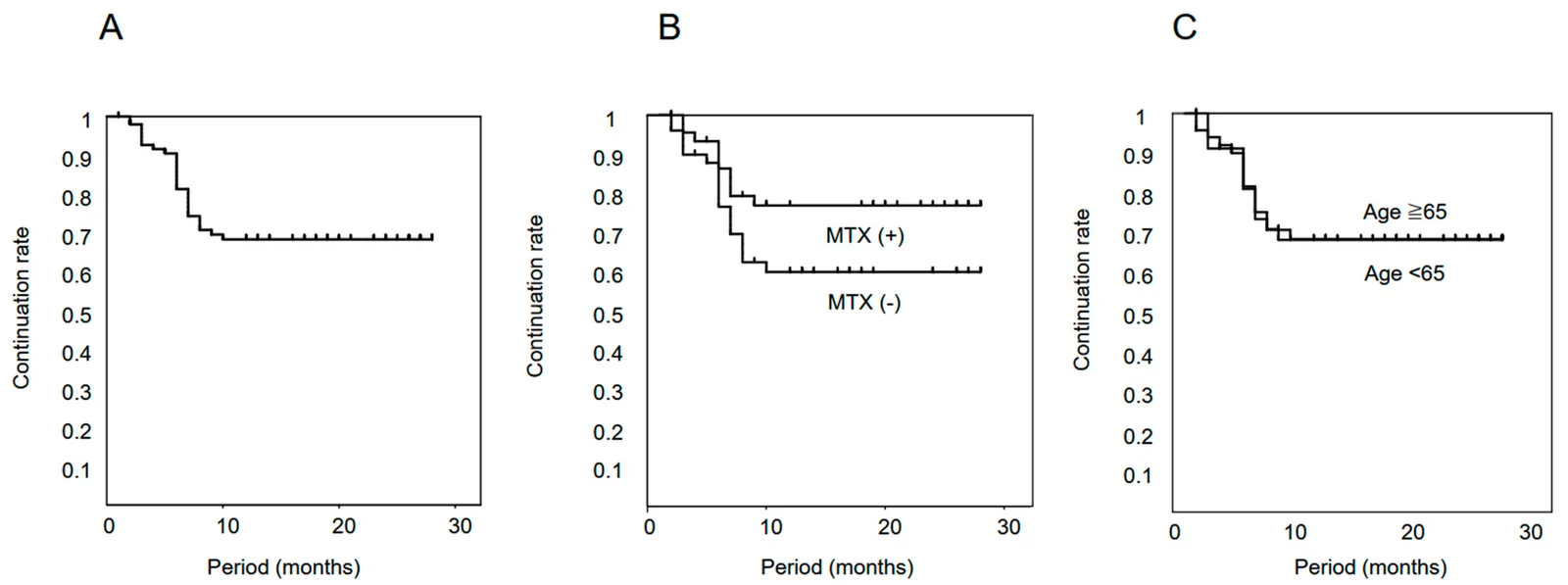
Continuation rate through 24 months in the overall cohort (**A**), according to MTX use (**B**), and according to age (<65 vs. ≥65 years) (**C**). MTX, methotrexate.

A logistic regression analysis showed that disease duration was significantly associated with remission at 24 weeks (*p* = 0.02347, OR = 0.95, 95% CI 0.909–0.993); however, there was no association with age (*p* = 0.48386, OR = 1.012, 95% CI 0.979–1.046), RF level (*p* = 0.86659, OR = 1.000, 95% CI 0.998–1.002), anti-CCP (*p* = 0.06629, OR = 1.003, 95% CI 1.000–1.005), MTX (*p* = 0.81862, OR = 1.014, 95% CI 0.897–1.147), or PSL (*p* = 0.21728, OR = 1.384, 95% CI 0.826–2.319) (Figure 5). The continuation rate was 68.4% at 24 months, including three biologic-free cases and one drug-free case (Figure 6A). Continuation rates did not differ significantly between the MTX-positive group (76.9%) and MTX-negative group (59.9%; log-rank test, *p* = 0.10055; Figure 6B) or between groups stratified by age at 65 years (log-rank test, *p* = 0.94732; Figure 6C). Adverse events occurred in 9% of patients and included fatigue (*n* = 2), skin rash (*n* = 4), pharyngitis (*n* = 1), pruritus (*n* = 1), and lower-limb cellulitis (*n* = 1).

## 4. Discussion

In this study, DAS28-CRP at 24 weeks correlated with anti-CCP in the MTX-positive group and disease duration in the MTX-negative and biologic-naïve groups. Remission at 24 weeks was correlated with disease duration. These findings suggest that ozoralizumab combined with MTX may be effective in biologic-naïve patients with high anti-CCP titers. In contrast, ozoralizumab may be effective in MTX-negative patients with early RA and a short disease duration. For example, a 51-year-old woman with an anti-CCP titer of 544 U/mL responded rapidly to ozoralizumab and achieved remission at 24 weeks. A 79-year-old biologic-naïve woman who was not receiving MTX and had a disease duration of 0.5 years also responded well to ozoralizumab and achieved remission at 24 weeks. Therefore, anti-CCP titer and disease duration may be predictive factors for ozoralizumab efficacy.

The rapid effect of ozoralizumab has been investigated in mouse models and attributed to its much smaller molecular size than the control [11]. In our cohort, some patients, particularly biologic-naïve patients receiving MTX, responded within 1 day. One such patient was a 74-year-old man with a disease duration of 2 years, stage II disease, class III functional status, MTX treatment at 12 mg/week, no PSL use, a CRP level of 3.53 mg/dL, an RF level of 269 U/mL, an anti-CCP titer of 19.5 IU/mL, an HAQ-DI score of 1.12, and a DAS28-CRP of 6.44. In contrast, a clinical trial of ozoralizumab showed that the serum trough level peaked at 16 weeks. Accordingly, several patients showed a delayed response to ozoralizumab up to 16 weeks after the initiation of treatment. A recent study of extending the ozoralizumab dosing interval to every 8 weeks reported a high rate of low disease activity [12]. However, extending the dosing interval was difficult in patients who had not achieved low disease activity by 24 weeks because of RA flares. Furthermore, the 2-year continuation rate did not differ significantly between patients ≥65 years of age and those <65 years of age. This study showed the safety of ozoralizumab when it was continued for two years in elderly patients.

Predictive factors of efficacy, including RF and anti-CCP status, have been reported for other biologic agents, such as abatacept, in patients with RA [13]. However, this is the first report to identify anti-CCP titer and disease duration as factors associated with the efficacy of the TNF blocker ozoralizumab in RA. These findings indicate that ozoralizumab may be more effective in seropositive patients than in seronegative patients, according to DAS28-CRP at 24 weeks. In our cohort, seronegative patients with low CRP levels showed insufficient responses. These findings suggest that ozoralizumab may be more effective in patients with anti-CCP-positive RA who are at risk of articular joint destruction. The continuation rate was higher in the MTX group than in the non-MTX group, although the difference was not statistically significant. Concomitant MTX may therefore remain important as anchor therapy during TNF blockade with ozoralizumab to inhibit joint destruction and the development of deformities.

By contrast, the OHZORA trials reported that neither RF nor anti-CCP status was associated with efficacy through 52 weeks [14]. Compared with the OHZORA trials, our real-world cohort had a lower MTX dose and baseline DAS28-CRP, as well as differences in age and disease duration. Furthermore, our study included switch patients (61%) and patients receiving ozoralizumab monotherapy, unlike the OHZORA trials. These findings suggest that an adequate MTX dose, such as >10 mg/week, is important for efficacy in biologic-naïve patients, regardless of RF or anti-CCP status. Ozoralizumab combined with MTX has also previously been reported to be more effective than ozoralizumab without MTX [15]. Therefore, concomitant MTX should be considered when evaluating the efficacy of ozoralizumab.

The limitations of this study include the lack of radiographic assessment of joint damage, long-term efficacy and safety data, its single-center design, the absence of a comparator group, relatively small subgroup sizes, potential confounding by indication, heterogeneous previous biologic therapies, heterogeneous MTX treatment, and the lack of analysis by Steinbrocker stage/class. Further long-term studies are needed to identify predictors of ozoralizumab efficacy.

## 5. Conclusions

The efficacy and safety of ozoralizumab were reconfirmed in 100 patients with RA in real-world clinical practice. Anti-CCP and disease duration are possible to be clinical predictors of ozoralizumab efficacy in patients with RA.

## Figures and Tables

**Table 1:** Baseline data of the treatment with Ozoralizumab.

*n*	100
Age	61.8 ± 14.1
Female/Male	87/13
Steinbrocker stage (I/II/III/IV)	16/43/16/25
D.D. (years)	10.7 ± 11.2
MTX (mg/w)	6.4 ± 2.3 (49/100)
PSL (mg/d)	0.38 ± 1.2 (10/100)
Naïve/Switch	39/61
DAS28(CRP)	4.38 ± 0.65
RF (IU/ml)	136.8 ± 226.1 (91/100)
CCP (U/ml)	140.2 ± 242.1 (74/100)

## Data Availability

The data in this study are included in this article and are available from the corresponding author upon reasonable request.

## Abbreviations

The following abbreviations are used in this manuscript

OZR	Ozoralizumab
TNF	Tumor necrosis factor
GC	Glucocorticoid
MTX	Methotrexate
csDMARDs	Conventional synthetic disease-modifying anti-rheumatic drugs
bDMARD	Biologic disease-modifying anti-rheumatic drugs
Anti-CCP	anti-cyclic citrullinated peptide
RF	rheumatoid factor
